# The Food Cure

**Published:** 1872-08

**Authors:** 


### THE FOOD CURE.

THE principles advocated in our book on "Health by Good Living," not only involve the prevention of disease by eating good, wholesome, nutritious, strength-giving food, well prepared, but go a step farther to advocate the cure of disease in general by the judicious use of food which comes upon our tables every day.

DIABETES, or the discharge daily of several quarts of sweet water or sugary urine, may be cured, and has been cured in two months, by living on five pints of milk a day, and a mutton chop.

